# A three-molecule score based on Notch pathway predicts poor prognosis in non-metastasis clear cell renal cell carcinoma

**DOI:** 10.18632/oncotarget.11849

**Published:** 2016-09-06

**Authors:** Zheng Liu, Qiang Fu, Hangcheng Fu, Zewei Wang, Le Xu, Huimin An, Yanfeng Li, Jiejie Xu

**Affiliations:** ^1^ Department of Biochemistry and Molecular Biology, School of Basic Medical Sciences, Fudan University, Shanghai, China; ^2^ Department of Urology, Ruijin Hospital, School of Medicine, Shanghai Jiaotong University, Shanghai, China; ^3^ Department of Orthopaedic Surgery, Shanghai Jiaotong University Affiliated Sixth People's Hospital, Shanghai, China

**Keywords:** clear cell renal cell carcinoma, Jagged1, Notch1, Hes1, prognostic factor

## Abstract

We constructed a three-molecule score based on the expression of Notch pathway molecules: Jagged1, intracellular Notch1 (ICN1) and Hes1 (JIH score). To assess prognostic value of the JIH score in non-metastasis clear cell renal cell carcinoma (ccRCC), we identified 467 patients who underwent nephrectomy during 2008-2009 as our study population. Immunohistochemistry was used to evaluate the expression of these three molecules. Cox regression models were applied to construct the JIH score, while Kaplan-Meier methods, multivariate analyses and nomogram were used to explore prognostic value of the JIH score. Our result confirmed that JIH score was an independent prognosticator for both overall survival (OS) and recurrence-free survival (RFS). Survival analyses showed that a higher JIH score indicated worse clinical outcomes (JIH score 3: 58.3% and 58.0% for 6-year OS and RFS, respectively; JIH score 0: 96.7% and 91.6% for 6-year OS and RFS, respectively). Nomograms based on JIH score and other conventional clinicopathological features had a better capability in predicting patients with pT1 stage disease for both OS and RFS (84.6% and 83.9%, respectively). The JIH score is a novel prognosticator representing activation of Notch pathway for non-metastasis ccRCC, and raises an alternative strategy for excavating potential biomarkers for signal pathways.

## INTRODUCTION

Renal cell carcinoma (RCC), according to the latest statistics, accounts for 61560 [1] estimated new cases in the USA, and for 66800 estimated new cases [2] in China. Most of RCC cases are clear cell RCC (ccRCC), with a nearly 80% proportion [3]. Thanks to innovative development of diagnosis and treatment, incident rate of metastatic RCC has been declined in past decades; in return, RCC confined for localized organ has been increased [4]. Tumor resection was normally recommended to patients with localized RCC, however, nearly 10% to 30% patients underwent surgery unavoidably suffered from local or distant recurrence and eventually ceased of terminal illness [5, 6]. Thus, selecting high-risk patients for more active surveillances and aggressive treatment is urgently needed.

Considering complex nature history of ccRCC, patients with similar clinical and pathological features may still experience distinct clinical outcome [7, 8]. However, contemporary prognostic models for RCC, such as UISS system [9], SSIGN score [10] and Leibovich score [11], are generally based on various clinicopathological factors. Molecular biomarkers may then provide additional information to clinicopathological factors, and help to improve their predictive accuracy [12–14].

In recent years, molecular and genetic mechanism of RCC has been further elucidated. Remarkably, nearly 60% incidence of ccRCC owed to the defect in the von Hippel-Lindau (VHL) gene [15]. Notch pathway controls various cellular process, including proliferation, apoptosis, and angiogenesis. [16] According to a recent study, Notch pathway was independent from VHL function in ccRCC, and its inhibition could restrain ccRCC growth both *in vitro* and *in vivo* [17]. Mammals have four Notch receptors (Notch1-4) with five Notch ligands (Jagged1, 2 and Delta-like 1, 3, 4). The transcriptional outputs of activated Notch pathways include basic helix-loop-helix factors of the hairy and enhancer of split (Hes) and Hes-related repressor protein (Hey) families [18]. In ccRCC, we previously reported that highly expressed Jagged1 and Notch1 predict poor outcomes [19, 20]. Furthermore, we also verified that Notch1 activation promotes ccRCC cell growth *in vitro* [21]. Nevertheless, some studies also revealed that Notch signaling plays as a tumor inhibitor in prostate cancer [22], pancreatic cancer [23] and liver cancer [24]. Studies in cervical cancer further suggest that Notch activation could restrain tumor formation initially, but could help tumor to progress in its late stage [25]. Depending on signal strength, timing and different cancer types, Notch signaling can play entirely different roles in tumorgenesis [26]. Because of this complexity, we here constructed a three-molecule score, named as JIH score, which consists of three important molecules in the Notch1 signaling pathway (ligand: Jagged1, active receptor: ICN1, output: Hes1). To further depict the clinical significance of Notch1 activation in ccRCC, we then investigated the prognostic value of the JIH score in association with clinical and pathological features of non-metastasis ccRCC patients.

## RESULTS

### Expression of Jagged1, ICN1 and Hes1 in ccRCC tumor tissue

**Figure 1:**
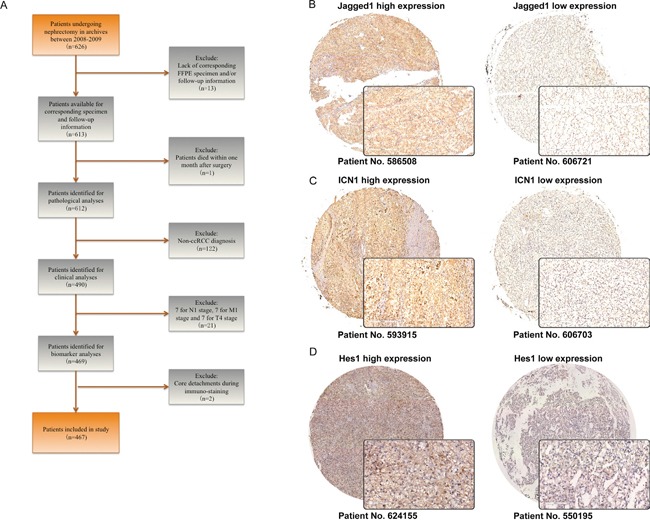
Expression of Jagged1, ICN1 and Hes1 in ccRCC tumor tissues **A.** Flowchart for study population enrollment. **B-D.** Representative immunohistochemistry staining pictures of (B) Jagged1, (C) ICN1, and (D) Hes1.

As shown in Figure 1B, 1C and 1D, Jagged1, ICN1 and Hes1 were expressed in tumor tissue respectively. Jagged1 and ICN1 were normally expressed in cytoplasm of tumor cell, while Hes1 was stained on tumor nuclear site. After evaluated by pathologists, cut-off values were determined as 75, 65 and 70 via minimum-p method for Jagged1, ICN1 and Hes1, respectively. Patient characteristics and associations with these three Notch markers were listed in Supplementary Table S1. All three markers were significantly related to Fuhrman grade, ECOG-PS, UISS score and Leibovich score. Indicating that these three markers may have positive correlation with tumor progression to some extent. Interestingly, only ICN1 expression had a statistical correlation with sarcomatoid features and Jagged1 expression failed to have a statistical correlation with SSIGN score. We blame these phenomena to the relative low population of subgroups within these factors (11 patients had sarcomatoid presented and 9 patients in ≥8 SSIGN score). Not surprisingly, all three markers were significantly correlated with death and recurrence, suggesting potential prognostic value of these three markers.

### Establishment of the JIH score

To explore correlation of Jagged1, ICN1 and Hes1, we first conducted multivariate Cox regression only including these three markers. Result was listed in Table 1, all three Notch markers were independent from each other. For both OS and RFS, coefficients of these three markers were all close to one, thus, we were able to treat these three markers without weighting each other. Hence, the JIH score was constructed by simply counting the number of altered three Notch markers. To determine a better and more efficient way to evaluate these three markers, c-indices were calculated. As shown in Table 1, combined Jagged1, ICN1 and Hes1 as a single factor, i.e. the JIH score, had a higher c-index than any of other three markers. We also applied in another way, taking Jagged1, ICN1 and Hes1 as three individual markers into Cox model at the same time. Although the c-index of this method was even higher than the JIH score, it failed to achieve a statistical significance. In addition, this method contributed three variables to the model, while the JIH score was only treated as a single variable. From the consideration of model stability and reasonable simplification, the JIH score was adopted throughout this study.

**Table 1:** Multivariate Cox regression and predictive accuracy analyses of Notch markers

	Multivariate Analyses			C-index	
**Overall survival**	**HR (95% CI)**	***P***	**Coefficient**		***P*†**
Jagged1 Expression (high vs. low*)	2.020 (1.125-3.935)	0.017	0.703	0.630	<0.001
ICN1 Expression (high vs. low*)	3.261 (1.747-6.952)	0.001	1.182	0.674	0.005
Hes1 Expression (high vs. low*)	2.596 (1.449-4.500)	0.002	0.954	0.650	<0.001
Jagged1, ICN1, Hes1 as 3 individuals	–	–	–	0.756	0.237
JIH Score	–	–	–	0.747	
**Recurrence free survival**					
Jagged1 Expression (high vs. low*)	1.765 (1.040-3.142)	0.035	0.568	0.614	<0.001
ICN1 Expression (high vs. low*)	2.472 (1.363-4.319)	0.003	0.905	0.649	0.005
Hes1 Expression (high vs. low*)	2.492 (1.436-4.116)	0.001	0.913	0.638	0.003
Jagged1, ICN1, Hes1 as 3 individuals	–	–	–	0.733	0.082
JIH Score	–	–	–	0.720	

Abbreviations: HR: Hazard Ratio; CI: confidence interval; ICN1: intracellular of Notch1; JIH score: Jagged1, ICN1 and Hes1 score.

* Reference group.

† JIH Score as reference groups.

All HR and 95%CI were calculated from 1000 bootstrap samples protected from overfitting.

### Correlation between the JIH score and patients' clinicopathological characteristics

As summarized in Table 2, age at surgery and follow-up time did not affect the JIH score. The JIH score have a strong positive correlation with T stage and Fuhrman grade, indicating it was related to tumor progression. However, we found tumor size and surgery type also presented positive correlations with the JIH score. Considering the connections between tumor size, surgery type and T stage, these phenomena were not surprising. ECOG-PS, UISS system, SSIGN score and Leibovich score were, as expected, positively and statistic significantly correlated with the JIH score, illustrating it may be an indicator for the tumor progression. Moreover, event number of death and recurrence also rose as the JIH score advanced, suggesting a potential prognostic value.

**Table 2:** Patient characteristics and associations with the JIH score

Factor	Patients		JIH score (number of altered Notch markers)				*P*
	No.	%	0 (n=135)	1 (n=177)	2 (n=106)	3 (n=49)	
Age at surgery (year)							0.650*
Median (IQR)	55 (46-63)		54 (47-61)	54 (45-64)	57 (48-63)	57 (49-64)	
Gender							0.051†
Male	329	70.4	100	130	68	31	
Female	138	29.6	35	47	38	18	
Surgery							<0.001†
Radical nephrectomy	238	51.0	54	86	63	35	
Partial nephrectomy	229	49.0	81	91	43	14	
Tumor size (cm)							0.039*
Median (IQR)	3.8 (2.5-5.0)		3.5 (2.5-5.0)	3.5 (2.5-5.0)	4.0 (3.0-6.0)	3.5 (3.0-6.0)	
T stage							<0.001‡
T1a	219	46.9	74	87	40	18	
T1b	112	24.0	35	43	25	9	
T2a	28	6.0	6	9	10	3	
T2b	4	0.9	0	1	2	1	
T3a	100	21.4	19	36	28	17	
T3b	4	0.9	1	1	1	1	
Fuhrman grade							<0.001‡
1	91	19.5	45	28	9	9	
2	218	46.7	62	87	52	17	
3	105	22.5	23	39	29	14	
4	53	11.3	5	23	16	9	
Tumor necrosis							0.740†
Absent	376	80.5	110	141	88	37	
Present	91	19.5	25	36	18	12	
Sarcomatoid							0.005†
Absent	456	97.6	135	174	100	47	
Present	11	2.4	0	3	6	2	
Lymphovascular invasion							0.107†
Absent	353	75.6	110	129	80	34	
Present	114	24.4	25	48	26	15	
ECOG-PS							0.001†
0	395	84.6	124	152	80	39	
≥1	72	15.4	11	25	26	10	
UISS							<0.001‡
Low risk	218	46.7	83	84	37	14	
Intermediate risk	222	47.5	50	84	57	31	
High risk	27	5.8	2	9	12	4	
SSIGN score							<0.001‡
0-3	345	73.9	111	135	68	31	
4-7	113	24.2	23	40	35	15	
≥8	9	1.9	1	2	3	3	
Leibovich score							<0.001‡
0-2	262	56.1	93	103	46	20	
3-5	163	34.9	39	58	47	19	
≥6	42	9.0	3	16	13	10	
Follow-up (month)							0.221*
Median (IQR)	73 (72-73)		73 (72-73)	73 (72-73)	73 (64-74)	72 (61-74)	
Death	57	12.2	4	14	20	19	<0.001†
Recurrence	65	13.9	6	19	21	19	<0.001†

Abbreviations: JIH score: Jagged1, ICN1 and Hes1 score; IQR: interquartile range; ECOG-PS: Eastern Cooperative Oncology Group performance status. UISS: UCLA Integrated Staging System; SSIGN: stage, size, grade and necrosis.

* Kruskal-Wallis H test

† Wilcoxon rank-sum test

‡ Spearman's rank correlation

### The JIH score was an independent prognosticator for ccRCC

We evaluated different combination strategies of Jagged1, ICN1 and Hes1 before, and selected the JIH score because of its highest c-index and relatively low variable number (Table 1). However, instead of evaluating combination methods within only Notch markers, we evaluated the predictive accuracy of the JIH score within other clinicopathological factors. As shown in Supplementary Table S2, Supplementary Table S3, and Table 3, JIH score still achieved a better prognostic accuracy than any other methods. Thus, we confirmed the JIH score as an ideal method in this study.

**Table 3:** Multivariate Cox regression analyses of clinicopathological features and the JIH score for overall survival and recurrence-free survival

Factor	HR (95% CI)	*P*
**Overall survival**	**C-index: 0.904**	
Tumor size (continuous, cm)	1.326 (1.188-1.480)	0.001
T stage		0.001
pT2 vs. pT1*	1.625 (0.569-4.642)	0.364
pT3 vs. pT1*	3.833 (1.844-7.967)	0.001
Fuhrman grade		0.001
3 vs. 1+2*	1.932 (0.959-3.891)	0.065
4 vs. 1+2*	6.353 (3.100-13.021)	0.001
Tumor necrosis (present vs. absent*)	3.661 (2.023-6.623)	0.001
Lymphovascular invasion (present vs. absent*)	3.013 (1.711-5.304)	0.001
JIH score		0.001
1 vs. 0*	1.857 (0.598-5.766)	0.284
2 vs. 0*	5.164 (1.743-15.298)	0.003
3 vs. 0*	15.432 (5.167-46.089)	0.001
**Recurrence-free survival**	**C-index: 0.889**	
Tumor size (continuous, cm)	1.345 (1.207-1.500)	0.001
T stage		0.003
pT2 vs. pT1*	0.993 (0.339-2.907)	0.989
pT3 vs. pT1*	2.946 (1.453-5.971)	0.003
Fuhrman grade		0.001
3 vs. 1+2*	2.455 (1.285-4.691)	0.007
4 vs. 1+2*	6.526 (3.348-12.720)	0.001
Tumor necrosis (present vs. absent*)	4.203 (2.419-7.302)	0.001
Lymphovascular invasion (present vs. absent*)	2.991 (1.734-5.159)	0.001
JIH score		0.001
1 vs. 0*	1.773 (0.690-4.551)	0.234
2 vs. 0*	3.872 (1.537-9.755)	0.004
3 vs. 0*	13.678 (5.239-35.196)	0.001

Abbreviations: JIH score: Jagged1, ICN1 and Hes1 score; HR: Hazard Ratio; CI: confidence interval.

All HR and 95%CI were calculated from 1000 bootstrap samples protected from overfitting.

* Reference group.

To inspect the prognostic independence of the JIH score, multivariate Cox regression analyses were conducted. To avoid instability of constructed model, features with a relative few populated subgroup, such as sarcomatoid features, were excluded from Cox models. ECOG-PS factor was also ruled out for its comparatively poor objectivity. As shown in Table 3, for both study endpoints, the JIH score was independent from other included clinicopathological factors. In both OS and RFS, the highest JIH score displayed an astonishing high risk compared with 0 JIH score (OS: HR=15.432, 95%CI=5.167-46.089, *P*=0.001; RFS: HR=13.678, 95%CI=5.239-35.196, *P*=0.001). Notably, in RFS, stage pT2 presented a close hazard ratio to stage pT1 (HR=0.993, 95%CI=0.339-2.907, *P*=0.989), which meant the hazard ratios of pT1 and pT2 were close and combination of pT1 and pT2 would be conducted in RFS during following analyses.

### The JIH score has a better prognosis value in early stage of ccRCC

**Figure 2:**
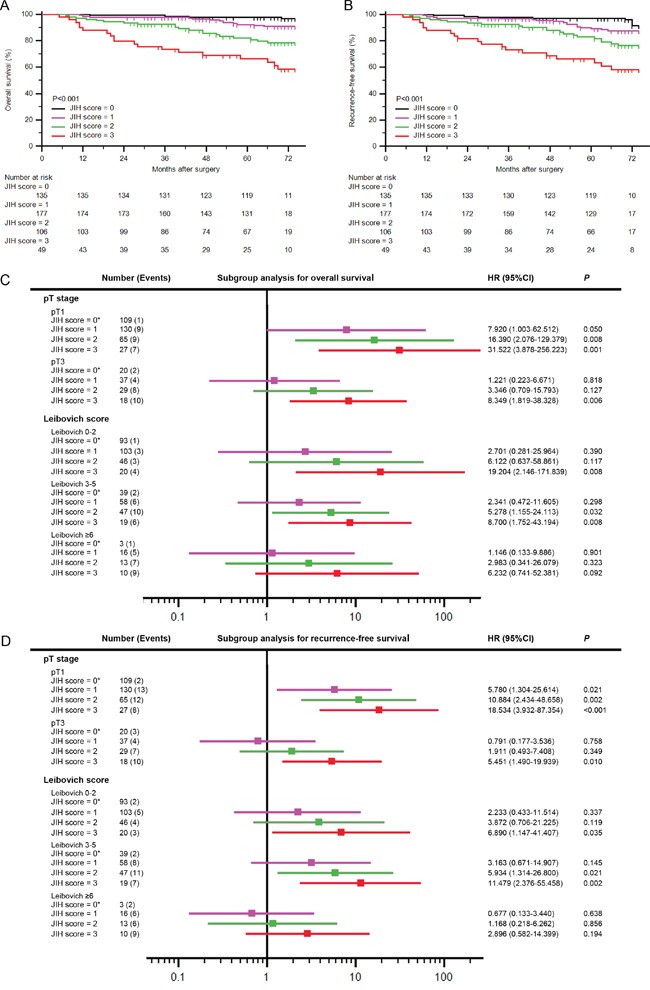
Prognostic power of the JIH score in diverse risk groups of ccRCC **A-B.** Kaplan-Meier curves in entire study population for (A) OS and (B) RFS according to the JIH score. **C-D.** Subgroup analyses of the JIH score in diverse pT stages and Leibovich score risk groups for (C) OS and (D) RFS.

To excavate further prognostic value of the JIH score, we conducted Kaplan-Meier survival analysis according to the JIH score. As illustrated in Figure 2A and 2B, higher JIH score indicated worse OS and RFS (*P*<0.001 and *P*<0.001, respectively). Patients with JIH score 3 had a 58.3% 6-year survival rate for OS and 58.0% for RFS while JIH score 0 patients had a much better 96.7% 6-year survival rate for OS and 91.6% for RFS.

To eliminate tumor progression stage bias on the JIH score, we conducted subgroup analysis within different prognosis systems. According to Kaplan-Meier survival analysis within pT1 patient group, higher JIH score indicated a worse OS and RFS (*P*<0.001) (Supplementary Figure S1). Though patients were staged as pT1, high JIH score would suggest a high hazard ratio compared to 0 JIH score for both OS and RFS (OS: JIH score 3: HR=31.522, 95%CI=3.878-256.223, *P*=0.001; RFS: JIH score 3: HR=18.534, 95%CI=3.932-87.354, *P*<0.001) (Figure 2C and 2D). Due to the population of pT2 patients was too few to run reliable calculation, we removed pT2 patients from subgroup analyses (pT2 patients=32, Table 2). In pT3 patient group, JIH score 1 and 2 failed to achieve statistical significance for stratification of hazard ratio for both OS and RFS (OS: JIH score 1: *P*=0.818, JIH score 2: *P*=0.127; RFS: JIH score 1: *P*=0.758, JIH score 2: *P*=0.349). JIH score 3 yet presented a strong prognostic ability in pT3 patients for both OS and RFS. Thus, the prognostic ability of the JIH score was independent of pT stage, and it had a better OS and RFS risk stratification in early stage patients.

The same findings were also observed in low risk patients according to diverse prognostic systems: Leibovich score, UISS system and SSIGN score (Figure 2, Supplementary Figure S1 and Supplementary Figure S2). UISS HR group and SSIGN≥8 group were removed from evaluation owing to relative few populations (UISS LR: n=27; SSIGN score≥8: n=9; Table 2). JIH score 3 predicted a worse hazard ratio in nearly all subgroups among various models (Figure 2 and Supplementary Figure S2). It was strange that the JIH score failed to present prognostic power in UISS LR group for neither OS nor RFS (Supplementary Figure S1 and Supplementary Figure S2). We suspected that relative few events in UISS LR group (OS: n=9, RFS: n=10; Supplementary Figure S2) decreased the stability in UISS LR analyses. Besides, UISS system was established on localized RCC patients instead of non-metastasis ccRCC patients in this study. These differences between study populations may also contribute the failure of JIH score predicting prognosis in UISS LR group.

### Nomograms based on the JIH score and predictive accuracy comparison

As a Notch pathway biomarker, the JIH score may have potential to improve prognostic ability combined with conventional clinicopathological features. Illustrating in Supplementary Figure S3A and S3C, two nomograms were constructed to predict OS and RFS at 1, 3 and 5 year after surgery. Calibration plot for each nomogram validated the predictive accuracy of corresponding nomograms (Supplementary Figure S3B and S3D). We also compared predictive accuracy of nomograms based on the JIH score with UISS, SSIGN and Leibovich system within pT1 patients. Results were listed in Table 4, the JIH score nomograms had higher c-indices and lower AICs, indicating a more accurate prognostic potential, especially among conventional low risk ccRCC patients.

**Table 4:** Comparison of prognostic accuracies of the Nomograms based on the JIH score, UISS, SSIGN and Leibovich scoring system among pT stage 1 population

**Overall survival**	**C-index**	***P* value**	**AIC**
Nomogram*	0.846		258.6
UISS	0.680	<0.001	285.8
SSIGN	0.691	<0.001	282.8
Leibovich	0.714	0.003	279.1
**Recurrence-free survival**	**C-index**	***P* value**	**AIC**
Nomogram*	0.839		339.8
UISS	0.708	0.003	373.1
SSIGN	0.719	<0.001	370.9
Leibovich	0.759	0.003	364.3

Abbreviations: JIH score: Jagged1, ICN1 and Hes1 score; UISS: UCLA Integrated Staging System; SSIGN: stage, size, grade and necrosis; AIC: Akaike's information criterion.

C-indices and AICs are calculated from 1000 bootstrap samples to protect from overfitting.

* Reference group

## DISCUSSION

We constructed an integrated algorithm of three key molecules originated from Notch signaling. A higher JIH score, which means a more activated Notch1 signaling, was a poor prognosticator for both OS and RFS of non-metastasis ccRCC patients. Further subgroups analyses revealed that the JIH score has a better prognostic value in low risk patients, and thus may serve as an indicator for selecting high-risk patients even they were regarded as low-risk tumors. As a biomarker, the JIH score could also add supplementary molecular pathway features to conventional clinicopathological factors, and improved their prognostic power. Nomograms based on the JIH score and other classical clinicopathological features were constructed. For both OS and RFS of pT1 ccRCC patients, nomograms containing the JIH score performed better than current clinicopathological-based prognostic models, such as UISS system, SSIGN score and Leibovich score. As the good applicability and visibility of nomogram, the JIH score nomogram might help clinicians to identity those high-risk patients even they were originally staged in a relatively low-risk group, especially JIH score 3 patients may have an astonishing high risk to experience worse clinical outcome. However, external validation in multiple clinical centers is necessary before the application of present findings.

As a well-known pleiotropic pathway, Notch signaling has various functions in pathologic process, including cancer [27, 28]. There are dozens of studies demonstrating the association between Notch activation and tumorigenesis [18]. In surprise, Notch signaling could act as a paradoxical role in various cancer types. The specific function of Notch pathway, either an oncogene or a tumor suppressor, depends on the specific tissue type and intensity of signaling [26]. For example, Notch in T cell acute lymphoblastic leukemia (T-ALL) was well characterized as an oncogene [29]; while in small cell lung carcinoma, Notch acted as a tumor suppressive role [30]. Moreover, even in the same histological tumor, Notch performed entirely different according to different stages of tumor process (in cervical caner) and different molecular subtypes (in medulloblasoma) [25, 31]. One reason for this paradoxical role of Notch signal may be the large number of downstream-targeted gene of Notch pathway. Besides Hes and Hey families, other frequent targets include MYC, cyclinD1, p21, BCL2, GATA3 and so on [32]. This target diversity results to highly complicated crosstalk between Notch and other cancer-related pathways, including MAPK, NF-κB, JNK, TGFβ, WNT, Hedgehog signaling [32] and, we previously reported, PI3K/Akt [21]. These extensively complicated molecular networks of Notch signaling in cancer doomed the unclarity of prognosticator studies that focus only in one biomarker, from the ligands, receptors and functional products, which floated upon the mighty molecular ocean of Notch [18].

Regarding RCC, the same obscure prognostic studies in Notch pathway also emerged. Notch signal was reported closely connected with VEGF pathway, which is a predominant progression pathway of RCC [33, 34]. Meanwhile, Notch is also believed to regulate tumor cell biology via HIF function, which is a downstream of VHL gene [35]. However, a recent study showed Notch regulates tumor cell in hypoxia independent of VHL [17]. Thus, it is undoubted that Notch pathway has a potential prognostic value in RCC patients. Unfortunately, most prognostic studies were based on one biomarker or one level of Notch signaling, leading to an elusive targeting strategy [36–40]. To avoid this kind of vague demonstration of Notch in RCC, we sought to combine three molecules at different levels of Notch signaling. Our previous works have proved that both Jagged1 and ICN1 expression are respectively independent adverse prognosticators for ccRCC [19, 20]. Besides, we found Hes1 protein expression were also increased in tumor tissues compared with normal renal tissues [21]. Since these three biomarkers were all validated in our former studies, we try to optimize Notch signaling in ccRCC prognostication using the JIH score. Indeed, the JIH score has higher c-indices than any single of these three biomarkers. The JIH score, at least partially, explored the prognostic value of Notch signaling in RCC.

Notably, several RCC prognostic studies related to molecular pathway also abandoned conventional one-molecular or one-level biomarker panels. Parker et al [13] constructed the BioScore system according to expression of B7-H1, survivin and Ki-67, which represent three different aspects of regulating cell death. These genetic heterogeneous molecules panels, although quite novel, was argued to “fail to add clarity” [41]. Haddad et al [14] adopted a multiple-level biomarker strategy in investigating mTOR pathway prognosticators in RCC. The author constructed a 5-biomarker panel composed of PI3K, PTEN, p-mTOR, p-4EBP1 and p-S6, which were all sprinkled throughout the mTOR pathway. This study, according to the author, has validated the prognostic value of mTOR pathway. However, as we previously reported, PI3K/Akt signaling was activated by Notch1 in ccRCC [21]. Since Notch signaling has various crosstalks with diverse signaling pathways, not only mTOR, the JIH score may present a more comprehensive biological scale in ccRCC.

Unfortunately, unlike mTOR pathway, there are still no FDA approved therapies for Notch pathway [32]. Researchers proposed that based on the highly complex nature function of Notch pathway, more selective and specific target among Notch pathway should be explicated to avoid poor specificity and side effects [18]. Simply targeting several Notch receptors, for example, dual Notch1 and Notch2 inhibition, caused side effects when treating patients [42]. Our study may provide alternative strategy to select detailed targets for specific cancer. At least in part, the JIH score may describe the therapeutic potential of Jagged1, ICN1 and Hes1 for non-metastasis ccRCC patients, rather than a single marker or markers at one single level.

A few limitations of this study should be noted. Firstly, although we used bootstraps as internal validation, external validation of multiple clinical centers is still needed. Secondly, as a natural limitation of IHC staining, we failed to measure biomarkers in a continuous manner, which is considered to be a more accurate parameter. Thirdly, some clinicopatholgical factors, such as sarcomatoid features, though are widely accepted to have prognostic association in ccRCC [3], were ruled out for its relatively low population. A larger scale of study population is also required to further validate the prognostic value of the JIH score.

In conclusion, the JIH score is an independent prognosticator for non-metastasis ccRCC, and has a stronger prognostic power in low-risk patients according to current prognostic models. Distinct from various prognostic studies in which only one marker or markers at a single level were assessed, the JIH score consists of three important molecules of Notch signaling pathway. The JIH score may also provide an alternative strategy for biomarker study on Notch signaling.

## MATERIALS AND METHODS

### Patients and follow-up

Approved by institution review board, 626 RCC patients undergoing nephrectomy between 2008 and 2009 in Zhongshan Hospital archive were recruited. Exclusion criterions were as follows: (1) lack of formalin fixed paraffin-embedded specimen or corresponding follow-up information; (2) died within one month after surgery; (3) pathologically diagnosed as non-ccRCC. Eventually, 467 cases were included in this study. A descriptive flowchart for patients selection was depicted in Figure 1A. All selected patients did not receive any anti-tumor therapy, and written consents were obtained from each patient. Patients were followed-up postoperatively with physical examinations, laboratory studies, chest imaging and abdominal ultrasounds or CT scans every six months for the first two years and annually thereafter. Endpoints were overall survival (OS) and recurrence-free survival (RFS), and were calculated from the date of surgery to the date of death from all causes and recurrence, respectively, or to the date of the last follow-up.

The median follow-up time was 73 months (range: 39-74). At the time of last follow-up, 57 patients (12.2%) had died of all causes; 65 patients (13.9%) had experienced recurrence. For each patient, clinicopathologic information were collected as follow: age, gender, surgery type, tumor size, T stage, Fuhrman grade, presence of tumor necrosis, sarcomatoid features, lymphovascular invasion and Eastern Cooperative Oncology Group performance status. All pathologic features were obtained by two experienced genitourinary pathologists (C. Zhai and Q. Fu) after reviewing original hematoxylin and eosin slides. Patients were staged via radiographic and postoperative pathologic reports, and were evaluated according to 2010 AJCC TNM classification. Since SSIGN system treated Fuhrman grade 1 and 2 as equal score [10], patients of grade 1 and grade 2 were combined during following analyses.

### Immunohistochemistry and evaluation

Tissue microarray construction and immunohistochemistry protocol were previously reported [43]. In brief, primary antibodies against human Jagged1 (ab7771, Abcam; dilution 1:300), ICN1 (ab8925, Abcam; dilution 1:400) and Hes1 (sc-25392, Santa Cruz; dilution 1:300) were applied. A semi-quantitative score was calculated for each case by multiplying the staining intensity (0, negative staining; 1, weak; 2, moderate; and 3, strong) and the percentage of stained cells (0%-100%) at each intensity level. Two experienced pathologists of urology (C. Zhai and Q. Fu) evaluated all staining blinded to the information of patients and the mean value of immunohistochemistry scores were adapted to avoid the inter-observer variability.

### Statistical analyses

Jagged1, ICN1 and Hes1 expression were dichotomized as low and high via minimum *P*-value methods. Associations between these three separate Notch markers expression and clincopathlogic factors were analyzed respectively with Chi-square test or Fisher's exact test for categorical variables, and Wilcoxon rank-sum test for continuous variables (Supplementary Table S1). Correlations between JIH score and other clincopathologic features were evaluated with Wilcoxon rank-sum test for unordered categorical variables, Spearman's rank correlation for ordered categorical variables and Kruskal-Wallis H test for continuous variables (Table 2). Survival curves were established via Kaplan-Meier methods, and were compared by log-rank test. Cox proportional hazard regression models were applied to conduct univariate and multivariate analyses, and were validated by 1000 bootstrap resamples to reduce overfit bias. Nomograms based on the JIH score and other clinicopathological features were constructed and their performance was illustrated within a calibration plot. The predictive accuracy of different prognostic models was calculated in Harrell concordance index (C-index), which ranges from 0.5 (no predictive power) to 1 (perfect predictive power). Akaike's information criterions (AIC) were also applied to compare the sufficiency of different models. Higher C-index and lower AIC represent a preferable model.

X-tile software, version 3.6.1 (Yale University School of Medicine, New Haven, CT) was used to determine cut-off value. Data were analyzed using SPSS 21.0 and Stata 13.0. Medcalc software was used to plot the survival curves and forest plots. R software (version 3.2.1, the ‘rms’ package) was used to build the nomograms. Reported recommendations for tumor marker prognostic studies (REMARK) list were self-checked throughout the study [44] (Supplementary Table S4).

## SUPPLEMENTARY MATERIALS FIGURES AND TABLES











## References

[1] Siegel RL, Miller KD, Jemal A (2015). Cancer statistics, 2015. CA Cancer J Clin.

[2] Chen W, Zheng R, Baade PD, Zhang S, Zeng H, Bray F, Jemal A, Yu XQ, He J (2016). Cancer statistics in China, 2015. CA Cancer J Clin.

[3] Ljungberg B, Bensalah K, Canfield S, Dabestani S, Hofmann F, Hora M, Kuczyk MA, Lam T, Marconi L, Merseburger AS, Mulders P, Powles T, Staehler M, Volpe A, Bex A (2015). EAU guidelines on renal cell carcinoma: 2014 update. Eur Urol.

[4] Pichler M, Hutterer GC, Chromecki TF, Jesche J, Kampel-Kettner K, Pummer K, Zigeuner R (2012). Renal cell carcinoma stage migration in a single European centre over 25 years: effects on 5- and 10-year metastasis-free survival. Int Urol Nephrol.

[5] Rini BI, Campbell SC, Escudier B (2009). Renal cell carcinoma. Lancet.

[6] Stewart GD, O'Mahony FC, Powles T, Riddick AC, Harrison DJ, Faratian D (2011). What can molecular pathology contribute to the management of renal cell carcinoma?. Nat Rev Urol.

[7] Sun M, Shariat SF, Cheng C, Ficarra V, Murai M, Oudard S, Pantuck AJ, Zigeuner R, Karakiewicz PI (2011). Prognostic factors and predictive models in renal cell carcinoma: a contemporary review. Eur Urol.

[8] Eichelberg C, Junker K, Ljungberg B, Moch H (2009). Diagnostic and prognostic molecular markers for renal cell carcinoma: a critical appraisal of the current state of research and clinical applicability. Eur Urol.

[9] Lam JS, Shvarts O, Leppert JT, Pantuck AJ, Figlin RA, Belldegrun AS (2005). Postoperative surveillance protocol for patients with localized and locally advanced renal cell carcinoma based on a validated prognostic nomogram and risk group stratification system. The Journal of urology.

[10] Frank I, Blute ML, Cheville JC, Lohse CM, Weaver AL, Zincke H (2002). An outcome prediction model for patients with clear cell renal cell carcinoma treated with radical nephrectomy based on tumor stage, size, grade and necrosis: the SSIGN score. The Journal of urology.

[11] Leibovich BC, Blute ML, Cheville JC, Lohse CM, Frank I, Kwon ED, Weaver AL, Parker AS, Zincke H (2003). Prediction of progression after radical nephrectomy for patients with clear cell renal cell carcinoma: a stratification tool for prospective clinical trials. Cancer.

[12] Rini B, Goddard A, Knezevic D, Maddala T, Zhou M, Aydin H, Campbell S, Elson P, Koscielny S, Lopatin M, Svedman C, Martini JF, Williams JA, Verkarre V, Radulescu C, Neuzillet Y (2015). A 16-gene assay to predict recurrence after surgery in localised renal cell carcinoma: development and validation studies. Lancet Oncol.

[13] Parker AS, Leibovich BC, Lohse CM, Sheinin Y, Kuntz SM, Eckel-Passow JE, Blute ML, Kwon ED (2009). Development and evaluation of BioScore: a biomarker panel to enhance prognostic algorithms for clear cell renal cell carcinoma. Cancer.

[14] Haddad AQ, Kapur P, Singla N, Raman JD, Then MT, Nuhn P, Buchner A, Bastian P, Seitz C, Shariat SF, Bensalah K, Rioux-Leclercq N, Sagalowsky A, Lotan Y, Margulis V (2015). Validation of mammalian target of rapamycin biomarker panel in patients with clear cell renal cell carcinoma. Cancer.

[15] Kim WY, Kaelin WG (2004). Role of VHL gene mutation in human cancer. J Clin Oncol.

[16] Leong KG, Karsan A (2006). Recent insights into the role of Notch signaling in tumorigenesis. Blood.

[17] Sjolund J, Johansson M, Manna S, Norin C, Pietras A, Beckman S, Nilsson E, Ljungberg B, Axelson H (2008). Suppression of renal cell carcinoma growth by inhibition of Notch signaling in vitro and in vivo. J Clin Invest.

[18] Ntziachristos P, Lim JS, Sage J, Aifantis I (2014). From fly wings to targeted cancer therapies: a centennial for notch signaling. Cancer Cell.

[19] Wu K, Xu L, Zhang L, Lin Z, Hou J (2011). High Jagged1 expression predicts poor outcome in clear cell renal cell carcinoma. Japanese journal of clinical oncology.

[20] An H, Zhu Y, Xu L, Chen L, Lin Z, Xu J (2015). Notch1 Predicts Recurrence and Survival of Patients With Clear-cell Renal Cell Carcinoma After Surgical Resection. Urology.

[21] Xu L, Zhu Y, Xu J, Wu K, Li J, Xu W, Liu H, Wang S, Yin H, Chen L, Wang G, Lin Z (2012). Notch1 activation promotes renal cell carcinoma growth via PI3K/Akt signaling. Cancer Sci.

[22] Whelan JT, Kellogg A, Shewchuk BM, Hewan-Lowe K, Bertrand FE (2009). Notch-1 signaling is lost in prostate adenocarcinoma and promotes PTEN gene expression. J Cell Biochem.

[23] Mullendore ME, Koorstra JB, Li YM, Offerhaus GJ, Fan X, Henderson CM, Matsui W, Eberhart CG, Maitra A, Feldmann G (2009). Ligand-dependent Notch signaling is involved in tumor initiation and tumor maintenance in pancreatic cancer. Clin Cancer Res.

[24] Wang M, Xue L, Cao Q, Lin Y, Ding Y, Yang P, Che L (2009). Expression of Notch1, Jagged1 and beta-catenin and their clinicopathological significance in hepatocellular carcinoma. Neoplasma.

[25] Talora C, Sgroi DC, Crum CP, Dotto GP (2002). Specific down-modulation of Notch1 signaling in cervical cancer cells is required for sustained HPV-E6/E7 expression and late steps of malignant transformation. Genes Dev.

[26] Mazzone M, Selfors LM, Albeck J, Overholtzer M, Sale S, Carroll DL, Pandya D, Lu Y, Mills GB, Aster JC, Artavanis-Tsakonas S, Brugge JS (2010). Dose-dependent induction of distinct phenotypic responses to Notch pathway activation in mammary epithelial cells. Proc Natl Acad Sci U S A.

[27] Artavanis-Tsakonas S, Rand MD, Lake RJ (1999). Notch signaling: cell fate control and signal integration in development. Science.

[28] Ranganathan P, Weaver KL, Capobianco AJ (2011). Notch signalling in solid tumours: a little bit of everything but not all the time. Nat Rev Cancer.

[29] Malyukova A, Dohda T, von der Lehr N, Akhoondi S, Corcoran M, Heyman M, Spruck C, Grander D, Lendahl U, Sangfelt O (2007). The tumor suppressor gene hCDC4 is frequently mutated in human T-cell acute lymphoblastic leukemia with functional consequences for Notch signaling. Cancer Res.

[30] Sriuranpong V, Borges MW, Ravi RK, Arnold DR, Nelkin BD, Baylin SB, Ball DW (2001). Notch signaling induces cell cycle arrest in small cell lung cancer cells. Cancer Res.

[31] Fan X, Mikolaenko I, Elhassan I, Ni X, Wang Y, Ball D, Brat DJ, Perry A, Eberhart CG (2004). Notch1 and notch2 have opposite effects on embryonal brain tumor growth. Cancer Res.

[32] Previs RA, Coleman RL, Harris AL, Sood AK (2015). Molecular pathways: translational and therapeutic implications of the Notch signaling pathway in cancer. Clin Cancer Res.

[33] Noguera-Troise I, Daly C, Papadopoulos NJ, Coetzee S, Boland P, Gale NW, Lin HC, Yancopoulos GD, Thurston G (2006). Blockade of Dll4 inhibits tumour growth by promoting non-productive angiogenesis. Nature.

[34] Patard JJ, Rioux-Leclercq N, Fergelot P (2006). Understanding the importance of smart drugs in renal cell carcinoma. Eur Urol.

[35] Gustafsson MV, Zheng X, Pereira T, Gradin K, Jin S, Lundkvist J, Ruas JL, Poellinger L, Lendahl U, Bondesson M (2005). Hypoxia requires notch signaling to maintain the undifferentiated cell state. Dev Cell.

[36] Li D, Masiero M, Banham AH, Harris AL (2014). The notch ligand JAGGED1 as a target for anti-tumor therapy. Front Oncol.

[37] Wang W, Yu Y, Wang Y, Li X, Bao J, Wu G, Chang H, Shi T, Yue Z (2014). Delta-like ligand 4: A predictor of poor prognosis in clear cell renal cell carcinoma. Oncol Lett.

[38] Sun S, Du R, Gao J, Ning X, Xie H, Lin X, Liu J, Fan D (2009). Expression and clinical significance of Notch receptors in human renal cell carcinoma. Pathology.

[39] Ai Q, Ma X, Huang Q, Liu S, Shi T, Zhang C, Zhu M, Zhang Y, Wang B, Ni D, Li H, Zheng T, Zhang X (2012). High-level expression of Notch1 increased the risk of metastasis in T1 stage clear cell renal cell carcinoma. PloS one.

[40] Sjolund J, Bostrom AK, Lindgren D, Manna S, Moustakas A, Ljungberg B, Johansson M, Fredlund E, Axelson H (2011). The notch and TGF-beta signaling pathways contribute to the aggressiveness of clear cell renal cell carcinoma. PloS one.

[41] Jonasch E (2009). Prognostic models: from the fates to the future. Cancer.

[42] Wu Y, Cain-Hom C, Choy L, Hagenbeek TJ, de Leon GP, Chen Y, Finkle D, Venook R, Wu X, Ridgway J, Schahin-Reed D, Dow GJ, Shelton A, Stawicki S, Watts RJ, Zhang J (2010). Therapeutic antibody targeting of individual Notch receptors. Nature.

[43] Liu Z, Liu Y, Xu L, An H, Chang Y, Yang Y, Zhang W, Xu J (2015). P2X7 receptor predicts postoperative cancer-specific survival of patients with clear-cell renal cell carcinoma. Cancer Sci.

[44] McShane LM, Altman DG, Sauerbrei W, Taube SE, Gion M, Clark GM, Statistics Subcommittee of the NCIEWGoCD (2005). Reporting recommendations for tumor marker prognostic studies. J Clin Oncol.

