# Supplementary material for: A three-molecule score based on Notch pathway predicts poor prognosis in non-metastasis clear cell renal cell carcinoma
Source: Oncotarget. 2016 Sep 6;7(42):68559–70. doi: 10.18632/oncotarget.11849 (PMC5356573; doi:10.18632/oncotarget.11849)
Supplement: Supplementary file 1 [file oncotarget-07-68559-s001.pdf]

A three-molecule score based on notch pathway predicts poor prognosis in non-metastasis clear cell renal cell carcinoma

SUPPLEMENTARY FIGURES AND TABLES

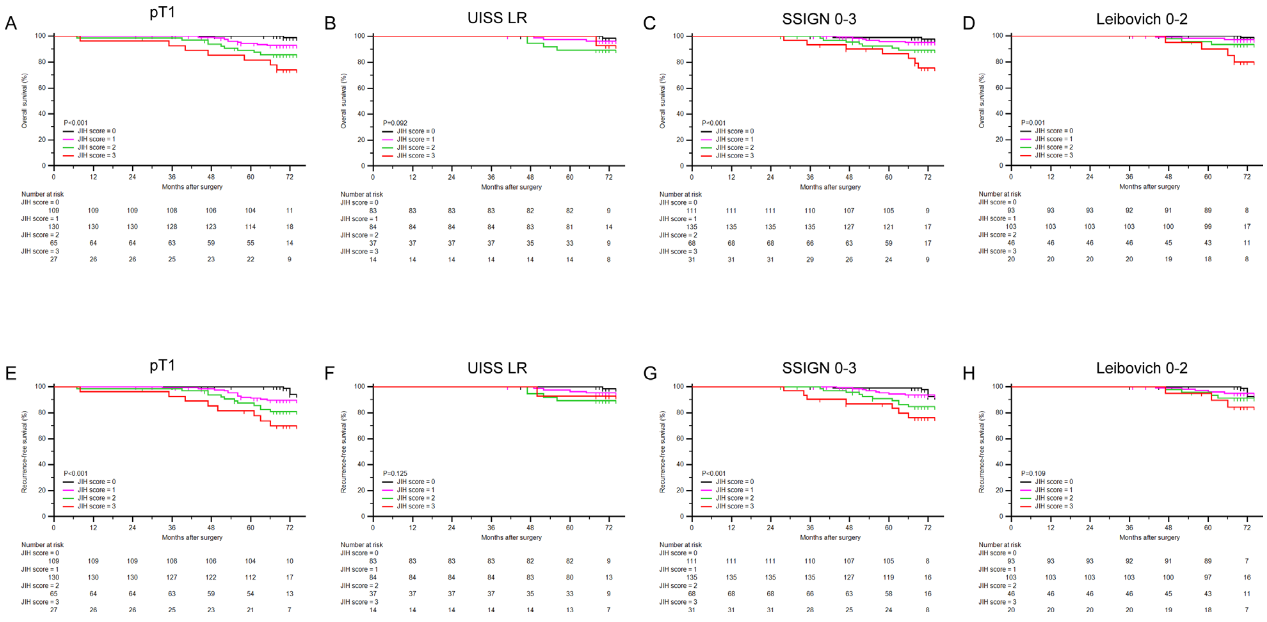

Supplementary Figure S1: Kaplan-Meier analyses ccRCC patients according to the JIH score in diverse early risk groups. A-D. Kaplan-Meier curves for OS of ccRCC patients according to JIH score in (A) pT1 stage, (B) UISS low risk, (C) SSIGN score 0-3, and (D) Leibovich score 0-2 patients. **E-H.** Kaplan-Meier curves for RFS of ccRCC patients according to the JIH score in (E) pT1 stage, (F) UISS low risk, (G) SSIGN score 0-3, and (H) Leibovich score 0-2 patients.

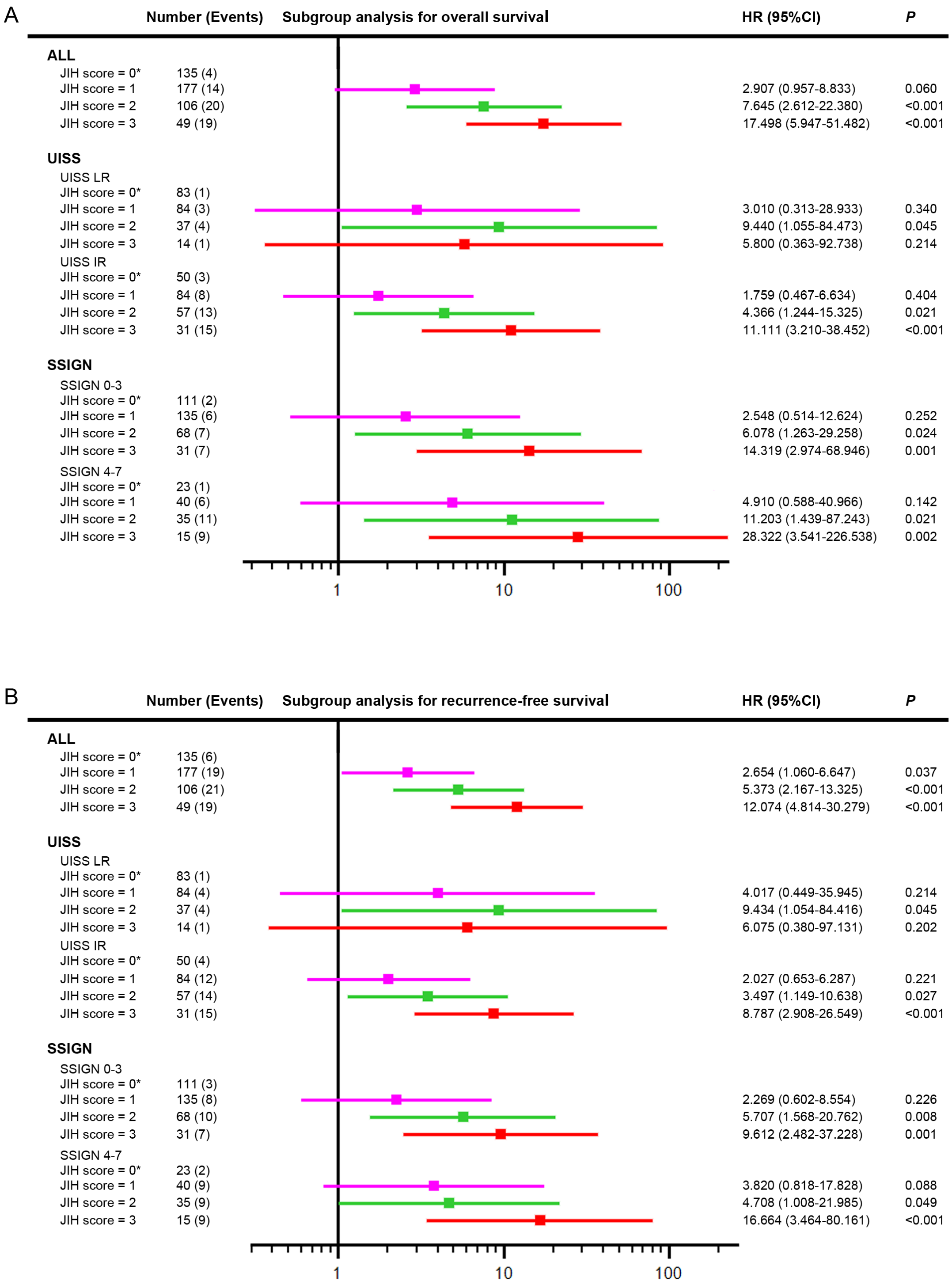

Supplementary Figure S2: Prognostic power of the JIH score in diverse risk groups of ccRCC. A-B. Subgroup analyses of the JIH score in all patients and in diverse UISS and SSIGN score risk groups for (A) OS and (B) RFS.

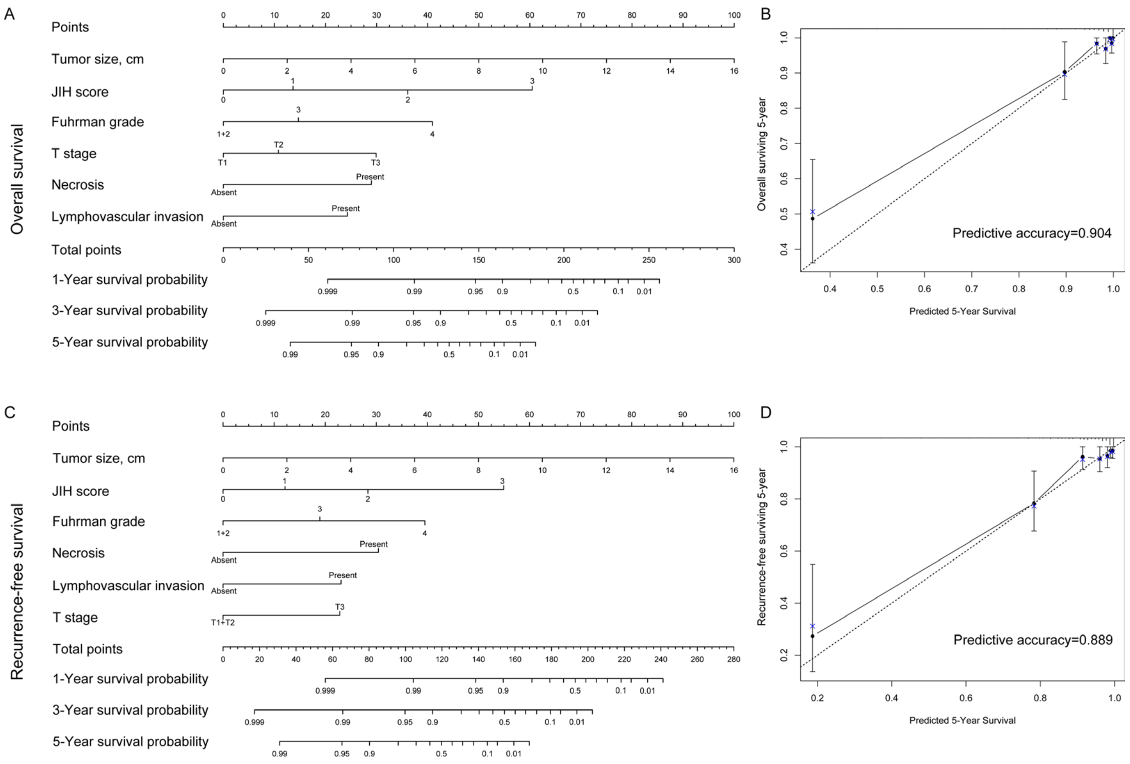

Supplementary Figure S3: Nomograms established based on the JIH score and other conventional clinicopathological factors. A. Nomogram predicting OS in non-metastatic ccRCC patients after surgery. Draw an upward vertical line from various factors to the point bar to calculate points. Based on the sums of points of various factors, draw a downward vertical line from the total points line to calculate OS. **B.** Calibration curves of the nomogram for 5-year OS. Dotted lines represent the performance of an ideal nomogram. Solid lines represent the performance of constructed nomogram. Error bars represent 95% CI. **C.** Nomogram for predicting RFS. **D.** Calibration curves of the nomogram for 5-year RFS.

Supplementary Table S1: Patient characteristics and associations with separated Notch markers

See Supplementary File 1

Supplementary Table S2: Univariate and multivariate Cox regression analyses of clinicopathological features and Notch markers for overall survival

See Supplementary File 2

Supplementary Table S3: Univariate and multivariate Cox regression analyses of clinicopathological features and Notch markers for recurrence-free survival

See Supplementary File 3

Supplementary Table S4: Adherence to REMARK guidelines

See Supplementary File 4
