# Supplementary material for: A three-molecule score based on Notch pathway predicts poor prognosis in non-metastasis clear cell renal cell carcinoma
Source: Oncotarget. 2016 Sep 6;7(42):68559–70. doi: 10.18632/oncotarget.11849 (PMC5356573; doi:10.18632/oncotarget.11849)
Supplement: Supplementary file 2 [file oncotarget-07-68559-s002.docx]

| Supplementary Table 1. Patient characteristics and associations with separated Notch markers | | | | | | | | | | | |
| --- | --- | --- | --- | --- | --- | --- | --- | --- | --- | --- | --- |
|  | **Patients** | | **Jagged1 Expression** | | | **ICN1 Expression** | | | **Hes1 Expression** | | |
| Factor | **No.** | **%** | **High (n=216)** | **Low (n=251)** | ***P*** | **High (n=204)** | **Low (n=263)** | ***P*** | **High (n=116)** | **Low (n=351)** | ***P*** |
| Age at surgery (year) |  |  |  |  | 0.302* |  |  | 0.930* |  |  | 0.112* |
| Median (IQR) | 55 (46-63) | | 56 (47-64) | 54 (46-62) |  | 56 (46-73) | 55 (46-63) |  | 57 (48-66) | 55 (46-62) |  |
| Gender |  |  |  |  | 0.145 |  |  | 0.242 |  |  | 0.179 |
| Male | 329 | 70.4 | 145 | 184 |  | 138 | 191 |  | 76 | 253 |  |
| Female | 138 | 29.6 | 71 | 67 |  | 66 | 72 |  | 40 | 98 |  |
| Surgery |  |  |  |  | 0.027 |  |  | 0.001 |  |  | 0.001 |
| Radical nephrectomy | 238 | 51.0 | 122 | 116 |  | 121 | 117 |  | 74 | 164 |  |
| Partial nephrectomy | 229 | 49.0 | 94 | 135 |  | 83 | 146 |  | 42 | 187 |  |
| Tumor size (cm) |  |  |  |  | 0.621* |  |  | 0.040* |  |  | 0.064* |
| Median (IQR) | 3.8 (2.5-5.0) | | 4.0 (3.0-5.0) | 3.8 (2.5-5.0) |  | 4.0 (3.0-5.9) | 3.5 (2.5-5.0) |  | 4.0 (3.0-6.4) | 3.8 (2.5-5.0) |  |
| T stage |  |  |  |  | 0.349 |  |  | 0.469 |  |  | <0.001 |
| T1a | 219 | 46.9 | 94 | 125 |  | 86 | 133 |  | 41 | 178 |  |
| T1b | 112 | 24.0 | 50 | 62 |  | 52 | 60 |  | 18 | 94 |  |
| T2a | 28 | 6.0 | 15 | 13 |  | 13 | 15 |  | 10 | 18 |  |
| T2b | 4 | 0.9 | 3 | 1 |  | 3 | 1 |  | 2 | 2 |  |
| T3a | 100 | 21.4 | 53 | 47 |  | 48 | 52 |  | 42 | 58 |  |
| T3b | 4 | 0.9 | 1 | 3 |  | 2 | 2 |  | 3 | 1 |  |
| Fuhrman grade |  |  |  |  | 0.010 |  |  | 0.001 |  |  | 0.012 |
| 1 | 91 | 19.5 | 32 | 59 |  | 28 | 64 |  | 13 | 78 |  |
| 2 | 218 | 46.7 | 100 | 118 |  | 90 | 128 |  | 52 | 166 |  |
| 3 | 105 | 22.5 | 50 | 55 |  | 53 | 52 |  | 36 | 69 |  |
| 4 | 53 | 11.3 | 34 | 19 |  | 33 | 20 |  | 15 | 38 |  |
| Tumor necrosis |  |  |  |  | 0.058 |  |  | 0.216 |  |  | 0.084 |
| Absent | 376 | 80.5 | 182 | 194 |  | 159 | 217 |  | 87 | 289 |  |
| Present | 91 | 19.5 | 34 | 57 |  | 45 | 46 |  | 29 | 62 |  |
| Sarcomatoid |  |  |  |  | 0.075 |  |  | 0.049 |  |  | 0.109 |
| Absent | 456 | 97.6 | 208 | 248 |  | 196 | 260 |  | 111 | 345 |  |
| Present | 11 | 2.4 | 8 | 3 |  | 8 | 3 |  | 5 | 6 |  |
| Lymphovascular invasion |  |  |  |  | 0.624 |  |  | 0.118 |  |  | 0.243 |
| Absent | 353 | 75.6 | 161 | 192 |  | 147 | 206 |  | 83 | 270 |  |
| Present | 114 | 24.4 | 55 | 59 |  | 57 | 57 |  | 33 | 81 |  |
| ECOG-PS |  |  |  |  | 0.048 |  |  | 0.014 |  |  | 0.035 |
| 0 | 395 | 84.6 | 175 | 220 |  | 163 | 232 |  | 91 | 304 |  |
| ≥1 | 72 | 15.4 | 41 | 31 |  | 41 | 31 |  | 25 | 47 |  |
| UISS |  |  |  |  | 0.004 |  |  | 0.014 |  |  | <0.001 |
| Low risk | 218 | 46.7 | 83 | 135 |  | 82 | 136 |  | 35 | 183 |  |
| Intermediate risk | 222 | 47.5 | 119 | 103 |  | 105 | 117 |  | 67 | 155 |  |
| High risk | 27 | 5.8 | 14 | 13 |  | 17 | 10 |  | 14 | 13 |  |
| SSIGN score |  |  |  |  | 0.137 |  |  | 0.043 |  |  | 0.001 |
| 0-3 | 345 | 73.9 | 151 | 194 |  | 142 | 203 |  | 71 | 274 |  |
| 4-7 | 113 | 24.2 | 59 | 54 |  | 55 | 58 |  | 41 | 72 |  |
| ≥8 | 9 | 1.9 | 6 | 3 |  | 7 | 2 |  | 4 | 5 |  |
| Leibovich score |  |  |  |  | 0.037 |  |  | 0.011 |  |  | <0.001 |
| 0-2 | 262 | 56.1 | 110 | 152 |  | 100 | 162 |  | 45 | 217 |  |
| 3-5 | 163 | 34.9 | 80 | 83 |  | 79 | 84 |  | 50 | 113 |  |
| ≥6 | 42 | 9.0 | 26 | 16 |  | 25 | 17 |  | 21 | 21 |  |
| Follow-up (month) |  |  |  |  | 0.920* |  |  | 0.129* |  |  | 0.007* |
| Median (IQR) | 73 (72-73) | | 73 (67-74) | 73 (72-73) |  | 73 (66-74) | 73 (72-73) |  | 72 (60-74) | 73 (72-73) |  |
| Death | 57 | 12.2 | 40 | 17 | <0.001 | 43 | 14 | <0.001 | 28 | 29 | <0.001 |
| Recurrence | 65 | 13.9 | 43 | 22 | 0.001 | 45 | 20 | <0.001 | 30 | 35 | <0.001 |
| Abbreviation: ICN1: intracellular Notch1; IQR: interquartile range; ECOG-PS: Eastern Cooperative Oncology Group performance status. UISS: UCLA Integrated Staging System; SSIGN: stage, size, grade and necrosis.  Fisher’s exact test was used when table data did not meet the requirement of Chi-square test.  * Wilcoxon rank-sum test. | | | | | | | | | | | |
