# Supplementary material for: A three-molecule score based on Notch pathway predicts poor prognosis in non-metastasis clear cell renal cell carcinoma
Source: Oncotarget. 2016 Sep 6;7(42):68559–70. doi: 10.18632/oncotarget.11849 (PMC5356573; doi:10.18632/oncotarget.11849)
Supplement: Supplementary file 3 [file oncotarget-07-68559-s003.docx]

| Supplementary Table 2. Univariate and multivariate Cox regression analyses of clinicopathological features and Notch markers for overall survival. | | | | | | |
| --- | --- | --- | --- | --- | --- | --- |
|  | **Univariate Analyses** | **Multivariate Analyses** | | | | |
|  |  | **Base model** | **Base model + Jagged1** | **Base model + ICN1** | **Base model + Hes1** | **Base Model + individual markers** |
| C-index |  | 0.854 | 0.880 | 0.885 | 0.873 | 0.903 |
| P-value |  | -- | 0.020 | 0.004 | 0.121 | 0.002 |
| Factor | HR (95% CI); ***P*** | HR (95% CI); ***P*** | HR (95% CI); ***P*** | HR (95% CI); ***P*** | HR (95% CI); ***P*** | HR (95% CI); ***P*** |
| Age at surgery (continuous by 5-year increment) | 1.113  (1.002-1.234);  0.036 | Not included | Not included | Not included | Not included | Not included |
| Gender (male vs. female*) | 1.610  (0.856-3.267);  0.164 | Not included | Not included | Not included | Not included | Not included |
| Tumor size (continuous, cm) | 1.495 (1.370-1.631); 0.001 | 1.310  (1.154-1.492);  0.001 | 1.347  (1.194-1.547);  0.001 | 1.305  (1.160-1.499);  0.001 | 1.320  (1.192-1.483);  0.001 | 1.343  (1.198-1.513);  0.001 |
| T stage | --; 0.001 | --; 0.001 | --; 0.001 | --; 0.001 | --; 0.001 | --; 0.001 |
| pT2 vs. pT1* | 5.270 (1.866-12.315); 0.001 | 2.109  (0.533-6.508);  0.147 | 1.640  (0.395-5.463);  0.329 | 2.232  (0.566-7.531);  0.135 | 1.675  (0.400-5.830);  0.335 | 1.543  (0.376-4.889);  0.449 |
| pT3 vs. pT1* | 4.563  (2.620-7.760);  0.001 | 5.228  (2.452-10.805);  0.001 | 4.697  (2.291-9.984);  0.001 | 5.228  (2.474-11.427); 0.001 | 3.838  (1.861-8.846);  0.001 | 3.857  (1.649-8.793);  0.001 |
| Fuhrman grade | --; 0.001 | --; 0.001 | --; 0.001 | --; 0.001 | --; 0.001 | --; 0.001 |
| 3 vs. 1+2* | 2.721  (1.353-5.419);  0.004 | 1.962 (0.951-3.983); 0.051 | 2.061  (1.008-4.208);  0.028 | 1.923  (0.953-3.751);  0.057 | 1.644  (0.753-3.633);  0.154 | 1.850  (0.829-4.133);  0.086 |
| 4 vs. 1+2* | 10.859  (5.836-20.348);  0.001 | 5.063  (2.298-11.612);  0.001 | 4.522  (1.939-9.934);  0.001 | 4.938  (2.312-10.924);  0.001 | 6.931  (3.180-14.746);  0.001 | 6.443  (2.954-15.425);  0.001 |
| Tumor necrosis (present vs. absent*) | 4.783  (2.773-8.207);  0.001 | 3.248  (1.714-5.995);  0.001 | 4.076  (2.102-7.553);  0.001 | 3.102  (1.627-5.865);  0.001 | 3.281  (1.747-6.271);  0.001 | 3.819  (1.912-7.799);  0.001 |
| Lymphovascular invasion (present vs. absent*) | 4.778  (2.869-8.314);  0.001 | 3.074  (1.626-6.437);  0.002 | 2.686  (1.307-5.458);  0.004 | 3.096  (1.644-6.322);  0.002 | 3.374  (1.725-6.540);  0.001 | 3.271  (1.621-6.896);  0.001 |
| Jagged1 expression (high vs. low*) | 3.016  (1.797-5.613);  0.001 | -- | 3.184  (1.745-6.554);  0.001 | -- | -- | 2.606  (1.399-5.382);  0.006 |
| ICN1 expression (high vs. low*) | 4.595  (2.612-8.750);  0.001 | -- | -- | 3.758  (2.036-7.606);  0.001 | -- | 2.956  (1.528-6.653);  0.004 |
| Hes1 expression (high vs. low*) | 3.593  (2.125-6.117);  0.001 | -- | -- | -- | 3.755  (2.020-7.308);  0.001 | 3.016  (1.507-6.360);  0.002 |
| JIH score | --; 0.001 | -- | -- | -- | -- | -- |
| 1 vs. 0* | 3.854  (1.121-16.135);  0.031 | -- | -- | -- | -- | -- |
| 2 vs. 0* | 10.329 (3.277-42.820);  0.001 | -- | -- | -- | -- | -- |
| 3 vs. 0* | 23.927 (7.286-107.233)  0.001 | -- | -- | -- | -- | -- |
| Abbreviation: HR: Hazard Ratio; CI: confidence interval; JIH score: Jagged1, ICN1 and Hes1 score.  * Reference group. All HR and 95%CI were calculated from 1000 bootstrap samples protected from overfitting. | | | | | | |
