# Supplementary material for: A three-molecule score based on Notch pathway predicts poor prognosis in non-metastasis clear cell renal cell carcinoma
Source: Oncotarget. 2016 Sep 6;7(42):68559–70. doi: 10.18632/oncotarget.11849 (PMC5356573; doi:10.18632/oncotarget.11849)
Supplement: Supplementary file 4 [file oncotarget-07-68559-s004.docx]

| Supplementary Table 3. Univariate and multivariate Cox regression analyses of clinicopathological features and Notch markers for recurrence-free survival. | | | | | | |
| --- | --- | --- | --- | --- | --- | --- |
|  | **Univariate Analyses** | **Multivariate Analyses** | | | | |
|  |  | **Base model** | **Base model + Jagged1** | **Base model + ICN1** | **Base model + Hes1** | **Base model + individual markers** |
| C-index |  | 0.849 | 0.869 | 0.873 | 0.866 | 0.888 |
| P-value |  | -- | 0.022 | 0.006 | 0.136 | 0.002 |
| Factor | HR (95% CI); ***P*** | HR (95% CI); ***P*** | HR (95% CI); ***P*** | HR (95% CI); ***P*** | HR (95% CI); ***P*** | HR (95% CI); ***P*** |
| Age at surgery (continuous by 5-year increment) | 1.055 (0.962-1.172); 0.273 | Not included | Not included | Not included | Not included | Not included |
| Gender (male vs. female*) | 1.226  (0.718-2.385);  0.561 | Not included | Not included | Not included | Not included | Not included |
| Tumor size (continuous, cm) | 1.476 (1.359-1.597); 0.001 | 1.314  (1.172-1.486);  0.001 | 1.353  (1.186-1.534);  0.001 | 1.322  (1.182-1.504);  0.001 | 1.327  (1.190-1.504);  0.001 | 1.355  (1.226-1.533);  0.001 |
| T stage | --; 0.001 | --; 0.001 | --; 0.001 | --; 0.001 | --; 0.005 | 0.003 |
| pT2 vs. pT1* | 3.304  (1.037-8.004);  0.008 | 1.274  (0.337-4.071);  0.575 | 1.014  (0.246-3.225);  0.815 | 1.260  (0.294-3.979);  0.608 | 0.958  (0.210-3.133);  0.906 | 0.855  (0.192-2.951);  0.974 |
| pT3 vs. pT1* | 3.449  (2.038-5.624);  0.001 | 3.785  (1.881-7.576);  0.001 | 3.438  (1.753-6.706);  0.001 | 3.770  (1.885-7.591);  0.002 | 2.841  (1.406-5.624);  0.003 | 2.875  (1.409-5.995);  0.002 |
| Fuhrman grade | --; 0.001 | --; 0.001 | --; 0.001 | --; 0.001 | --; 0.001 | 0.001 |
| 3 vs. 1+2* | 3.466  (1.848-6.606);  0.001 | 2.535  (1.288-5.160);  0.004 | 2.537  (1.320-5.150);  0.002 | 2.542  (1.220-4.865);  0.002 | 2.179  (1.089-4.259);  0.019 | 2.351  (1.148-4.674);  0.015 |
| 4 vs. 1+2* | 12.554  (6.945-23.127);  0.001 | 6.278  (2.992-13.640);  0.001 | 5.212  (2.430-10.730);  0.001 | 5.824  (2.793-12.195);  0.001 | 8.053  (3.721-17.322);  0.001 | 6.862  (3.200-14.880);  0.001 |
| Tumor necrosis (present vs. absent*) | 5.043  (2.989-8.265);  0.001 | 3.438  (1.879-6.373);  0.001 | 4.238  (2.300-8.101);  0.001 | 3.480  (1.927-6.488);  0.001 | 3.611  (2.081-6.449);  0.001 | 4.289  (2.351-7.917);  0.001 |
| Lymphovascular invasion (present vs. absent*) | 4.349  (2.622-7.192);  0.001 | 2.643  (1.416-5.008);  0.004 | 2.382  (1.218-4.509);  0.009 | 2.798  (1.581-4.894);  0.001 | 2.846  (1.598-5.058);  0.001 | 3.071  (1.647-5.954);  0.001 |
| Jagged1 expression (high vs. low*) | 2.487  (1.495-4.100);  0.001 | -- | 2.748  (1.448-5.181);  0.001 | -- | -- | 2.228  (1.262-4.195);  0.006 |
| ICN1 expression (high vs. low*) | 3.287  (2.022-5.579);  0.001 | -- | -- | 2.889  (1.770-5.186);  0.001 | -- | 2.425  (1.289-4.600);  0.003 |
| Hes1 expression (high vs. low*) | 3.235  (2.050-5.254);  0.001 | -- | -- | -- | 3.586  (1.948-6.733);  0.001 | 3.089  (1.701-5.732);  0.001 |
| JIH score | --; 0.001 | -- | -- | -- | -- | -- |
| 1 vs. 0* | 2.936  (1.161-9.689);  0.023 | -- | -- | -- | -- | -- |
| 2 vs. 0* | 6.013  (2.387-19.965);  0.001 | -- | -- | -- | -- | -- |
| 3 vs. 0* | 13.558 (5.186-49.600);  0.001 | -- | -- | -- | -- | -- |
| Abbreviation: HR: Hazard Ratio; CI: confidence interval.  * Reference group. All HR and 95%CI were calculated from 1000 bootstrap samples protected from overfitting._­­­_ | | | | | | |
